# Pediatric H3 G34-mutant diffuse hemispheric glioma: clinical, imaging and molecular prognostic factors, *MGMT* expression, and temozolomide response

**DOI:** 10.1007/s00401-026-02992-w

**Published:** 2026-03-02

**Authors:** Dana Tlais, Qunyu Zhang, Jordan T. Roach, Christopher L. Tinkle, Tong Lin, Xiaoyu Li, Ayatullah Mostafa, Daniel C. Moreira, Rene Y. McNall-Knapp, Sarah Z. Rush, Brian H. Le, Sara Sinno, Apeksha Ramnarayan, Kevin F. Ginn, Sonia Partap, Arzu Onar-Thomas, Larissa V. Furtado, Asim K. Bag, Jason Chiang

**Affiliations:** 1 Department of Oncology, St. Jude Children’s Research Hospital, Memphis, USA; 2 Department of Pathology, St. Jude Children’s Research Hospital, 262 Danny Thomas Place, MS 250, Memphis, TN 38105-3678 USA; 3 Department of Developmental Neurobiology, St. Jude Children’s Research Hospital, Memphis, USA; 4 Department of Radiation Oncology, St. Jude Children’s Research Hospital, Memphis, USA; 5 Department of Biostatistics, St. Jude Children’s Research Hospital, Memphis, USA; 6 Department of Diagnostic Imaging, St. Jude Children’s Research Hospital, Memphis, USA; 7 Department of Global Pediatric Medicine, St. Jude Children’s Research Hospital, Memphis, USA; 8 Department of Pediatrics, University of Oklahoma Health Sciences Center, Norman, USA; 9 Department of Pediatrics, Akron Children’s Hospital, Akron, USA; 10 Department of Pathology, University of North Carolina School of Medicine, Chapel Hill, USA; 11 Department of Pathology and Laboratory Medicine, American University of Beirut Medical Center, Beirut, Lebanon; 12 Department of Pathology and Laboratory Medicine, UT Health San Antonio, San Antonio, USA; 13 Department of Pediatrics, Children’s Mercy, Kansas City, USA; 14 Department of Neurology, Stanford University, Stanford, USA

**Keywords:** H3 G34-mutant diffuse hemispheric glioma, *MGMT*, Temozolomide, Prognostic factors, Treatment outcomes

## Abstract

Previous studies have demonstrated poor outcomes in pediatric patients with H3 G34-mutant diffuse hemispheric glioma (DHG). However, the biological basis for this therapeutic resistance remains poorly understood. Furthermore, the effectiveness of temozolomide (TMZ) and the role of surgery in pediatric patients remain uncertain. Therefore, we performed a multi-institutional retrospective analysis of the clinical, imaging, and molecular characteristics of 36 pediatric (≤ 18 years) patients with newly diagnosed H3 G34-mutant DHG. The median age of the cohort was 14 years (8–18 years). The median progression-free survival (PFS) was 0.7 years (95% CI 0.4–1.2 years), and the median overall survival (OS) was 1.8 years (95% CI 1.1–3.2 years). Gross total resection (GTR) was associated with improved PFS (*p* = 0.0046). Infiltration of three or more brain lobes (gliomatosis cerebri) was noted in 22.6% (7/31) of patients at presentation. Twenty-one patients (58.3%) received frontline TMZ and had improved PFS (*p* = 0.0049) compared to those who did not. Low *MGMT* expression was associated with better PFS (*p* = 0.0039) and better OS (*p* < 0.0001). In pediatric DHG, gene body/intronic CpG methylation, rather than promoter methylation, correlated with *MGMT* expression (*p* < 0.0001). *MGMT* promoter methylation was not significantly associated with PFS or OS. *PDGFRA* alterations (*n* = 13) were associated with inferior OS (*p* = 0.0035). Post-radiation local (± distant) recurrence occurred in 81.0% (17/21) of patients. Our findings reaffirm the dismal outcomes of pediatric H3 G34-mutant DHG, which exhibits radiation resistance, frequent widespread disease, and a novel mechanism of *MGMT* regulation. Our data support the use of frontline TMZ in pediatric patients and underscore the importance of GTR when feasible.

**Supplementary Information:**

The online version contains supplementary material available at https://doi.org/10.1007/s00401-026-02992-w.

## Key points

**Table:** 

Surgery, temozolomide use, and molecular status correlated with outcomes in pediatric DHG.
*MGMT* expression, not promoter methylation, correlated with survival.
Most treatment failures occurred within the high-dose radiation field.

## Introduction

Pediatric-type diffuse high-grade gliomas (pHGGs) are molecularly diverse and fatal central nervous system (CNS) tumors [32, 38]. Nearly 30% of pHGGs occurring in the cerebral hemispheres of older adolescents and young adults have a recurrent alteration in *H3-3A* at amino acid 34 (glycine to arginine/valine), a defining molecular feature of H3 G34-mutant diffuse hemispheric glioma (DHG) [21, 27, 36, 46]. In addition to harboring concomitant loss-of-function mutations in *TP53* and *ATRX*, H3 G34-mutant DHG frequently exhibits *PDGFRA* amplification and activating mutations [5]. Homozygous deletion of *CDKN2A/B* is also recognized as an important feature of this rare brain tumor [45]. There remains a paucity of cohorts with granular clinical annotation despite new insights into the unique developmental origins of H3 G34-mutant DHG [3, 5, 16, 21, 23, 25, 34]. The limited literature on pediatric H3 G34-mutant DHG has further hindered the identification of molecular prognostic markers and patterns of treatment failure in this population.

Treatment regimens for H3 G34-mutant DHG, aside from surgery and focal radiation therapy (RT), are limited. The incorporation of temozolomide (TMZ) is a common approach, as extrapolated from data in adults with newly diagnosed high-grade glioma (HGG) [19, 39], as well as some pediatric data [10, 20]. There is considerable variability in frontline medical therapy for these patients with consistently poor outcomes [31]. Outcomes appear particularly poor in pediatric patients, with shorter survival reported [12, 13, 47]. In addition, while O^6^-methylguanine-DNA methyltransferase (*MGMT)* promoter methylation status is a valuable molecular marker in adults with newly diagnosed HGG, where a methylated status confers a survival advantage and predicts a response to TMZ [19, 39], pediatric data are inconsistent. For example, large pediatric studies, including ACNS0822 [26] and the HERBY trial [28], refute the prognostic and predictive role of *MGMT* promoter methylation. The importance of this marker in H3 G34-mutant DHG has also been explicitly explored, with conflicting data. Some reports suggest a critical prognostic and/or predictive role [21, 23], while others indicate a lack of such a central role [43]. The heterogeneous data on H3 G34-mutant DHG prompted us to question whether a biological difference exists between the adult and pediatric populations that drives the survival differences and whether the mechanism of *MGMT* regulation differs in children compared to adults.

To address these critical questions, we studied a pediatric cohort of patients with H3 G34-mutant DHG. Here, we report a comprehensive evaluation of clinical, imaging, and molecular features in a multi-institutional cohort of pediatric patients with newly diagnosed H3 G34-mutant DHG. We also assess therapeutic modalities and their association with treatment response and survival outcomes. To our knowledge, our multi-institutional study represents the largest pediatric cohort with detailed clinical annotations and extended follow-up for patients diagnosed with H3 G34-mutant DHG. It is the first to describe unique aspects of pediatric H3 G34-mutant DHG and to characterize a novel mechanism of *MGMT* regulation in pediatrics. Our study offers data-driven recommendations for frontline surgical and medical therapy.

## Materials and methods

### Study cohort

We conducted a retrospective chart review on 36 patients with newly diagnosed H3 G34-mutant DHG between 2006 and 2024. Twenty-three patients were treated at St. Jude Children’s Research Hospital, and 13 patients were treated at collaborating institutions (The University of Oklahoma Health Sciences Center, Akron Children’s Hospital, The University of North Carolina School of Medicine, The University of Texas Health San Antonio, Children’s Mercy Kansas City, American University of Beirut Medical Center, and Lucile Packard Children’s Hospital at Stanford). Collected data included patient demographics, tumor location, molecular alterations, surgical resection status, treatment details, and survival outcomes. Frontline TMZ use was defined as the receipt of at least five doses of TMZ during RT and/or at least one completed adjuvant cycle of TMZ immediately following RT (any dose). Institutional review board approval was obtained at St. Jude (IRB# 19-0338, 22-1199, 24-1699) and at external institutions based on local guidelines.

### Whole genome sequencing

Genomic DNA was extracted from snap-frozen tumor tissue using an automated Maxwell RSC Instrument (Promega), as previously described [6, 15, 18]. DNA quality was assessed on a 4200 TapeStation (Agilent). Paired-end sequencing was conducted on the Illumina HiSeq platform with a 100- or 125-bp read length or NovaSeq with a 150-bp read length. Sequencing results were analyzed using an institutionally established pipeline for alignment and calling of single-nucleotide variants (SNVs), insertions, and deletions (indels). Alterations were annotated and ranked by putative pathogenicity using a workflow named "medal ceremony" and subsequently manually reviewed [48].

### Whole exome sequencing

Genomic DNA was extracted from formalin-fixed paraffin-embedded (FFPE) tissue using a QIAamp DNA FFPE Tissue Kit (Qiagen), as previously described [6, 8, 15, 18]. The genomic libraries were generated using the SureSelectXT kit (Agilent Technologies), followed by exome enrichment using the SureSelectXT Human All Exon V8 bait set (Agilent Technologies). The resulting exon-enriched libraries were subjected to the paired-end, 100-cycle sequencing performed on a NovaSeq X Plus (Illumina). For variant calling, the data were processed using GATK [42] (v.4.5.0) following the GATK Best Practices and manually inspected for accuracy.

### Transcriptome sequencing (RNA-seq)

Total RNA was extracted from FFPE slides using a PureLink™ FFPE RNA Isolation Kit (Thermo Fisher Scientific) according to the manufacturer’s instructions, as previously described [6–9, 24]. The RNA-seq data were aligned to the human reference genome (build hg38) using STAR8 and normalized using edgeR [29] (v.4.2.2) in R 4.4.1.

### Methylation array analysis

Genomic DNA was extracted as described above and subjected to genome-wide methylation profiling on a MethylationEPIC v2.0 BeadChip platform, as previously described [6–9, 18, 24]. Copy-number variation (CNV) analysis was performed using conumee, as previously described [14]. A reference control set was created using non-neoplastic brain tissue samples processed on the same platform to establish a baseline diploid state. Segmentation was performed using the Circular Binary Segmentation algorithm implemented in conumee. Automated CNV calling was performed by mapping genomic segments to the coordinates of target genes. A log_2_ intensity ratio > 0.9 and < − 1.2 was used as the cutoff for amplification and homozygous deletion, respectively. *MGMT* promoter methylation was assessed using MGMT-STP27, as previously described [1, 2]. The normalized *β* values and *M* values of each CpG site were calculated using ChAMP [40] 2.29.1 in R 4.4.1 with the default settings.

For the correlation analysis between CpG methylation of the *MGMT* locus and *MGMT* mRNA expression levels, the correlation coefficients were analyzed using the Spearman method between the normalized *β* values or *M *values of the CpG sites located in the *MGMT* region and the normalized counts of *MGMT* transcripts. CpG sites with absolute correlation coefficients greater than 0.70 were considered significantly correlated. Multiple testing was controlled by False Discovery Rate (FDR) by default, applying the Benjamini–Hochberg (BH) procedure to calculate adjusted *p* values.

### Histopathology review and immunohistochemistry

Hematoxylin and eosin-stained 5 μm sections of FFPE tissue specimens of all tumor samples were centrally reviewed by a board-certified neuropathologist specialized in pediatric CNS tumors (JC) to confirm the diagnosis. The following antibodies were used on 5 μm FFPE tissue sections: p53 (Zeta Corp, Z2029M, clone DO-7, 1:200) and ATRX (Sigma, HPA001906, 1:600).

### Imaging review

For patients with available preoperative imaging (*n* = 31), the baseline brain MRI (before any therapy) and all the subsequent scans were reviewed by a neuroradiologist (AKB) until evidence of recurrence or progression was found. Each scan consisted of pre- and postcontrast T1-weighted, T2-weighted, fluid-attenuated inversion recovery (FLAIR), and diffusion-weighted sequences. Blood-sensitive sequences, either gradient-recalled echo (GRE) or susceptibility-weighted imaging (SWI), were used to evaluate intratumoral hemorrhage when available. Non-contrast CT scans obtained at presentation were also reviewed for hemorrhage. At presentation, scans were categorically evaluated for (a) location of the tumor, midline vs. lobar vs. multilobar; (b) patterns of tumor margins, well defined vs. infiltrative; (c) focality of the tumor, focal vs. multifocal; (d) presence of hemorrhage; (e) presence of enhancement; (f) patterns of diffusion restriction; and (g) presence of metastasis throughout the neuraxis. A focal lesion was defined as a single lesion with well-defined margins on postcontrast T1-weighted sequence with or without proportionate T2 abnormality suggestive of peritumoral edema surrounding the T1-enhancing lesion. An infiltrative lesion is defined as a lesion with either poorly defined T1-enhancing margins, abnormal disproportionate T2 signal around the T1-enhancing lesion, or an ill-defined margin in tumors with no enhancing component. Infiltrative lesions were further evaluated for contiguous multiple lobar involvement. Progression was defined as the development of a new lesion or worsening of a previously treated lesion and was evaluated for either local or distant progression, as defined below. The focality of the progression was also assessed, as was the extent of progression with respect to crossing midline and the presence of subventricular spread. A gross total resection (GTR) was defined as a clean, well-defined surgical margin with no evidence of residual tumor on any sequence. A near-total resection (NTR) was defined as when the imaging appearance across all sequences resembled a focal residual tumor (~ 5% of the initial mass), predominantly along well-defined, clean surgical margins. A subtotal resection (STR) was defined as a substantial residual tumor. Gliomatosis cerebri was defined as tumor involvement of at least three lobes of the brain.

### Radiation dosimetry and pattern of failure analysis

For radiotherapy dosimetric assessment, complete CT datasets from the radiation treatment plans were transferred to MIM software (MIM Software Inc., Cleveland, OH), and composite radiation dose data were assembled. MRI scans at progression were co-registered to CT datasets using standard vendor-supplied software, and the anatomic tumor extent at progression was manually delineated based on T2-weighted FLAIR and T1 postcontrast MRI abnormalities. Dose-volume histograms were calculated for the progression volumes, and progression with respect to radiation dose distribution was categorized as previously done for adult and pediatric patients with HGG [4, 41].

### Statistical analysis

Progression-free survival (PFS) was defined as the time interval from the date of first surgery to the date of disease progression or death from any cause or to the date of last contact for patients without progression. Overall survival (OS) was defined as the time interval from the date of first surgery to the date of death from any cause or to the date of last contact for survivors. The Kaplan–Meier (KM) method was used to estimate survival outcomes. The difference between survival curves was compared by the log-rank test. Fisher’s exact tests were used to investigate associations between categorical variables.

## Results

### Clinical, imaging, and molecular characteristics

**Fig. 1:**
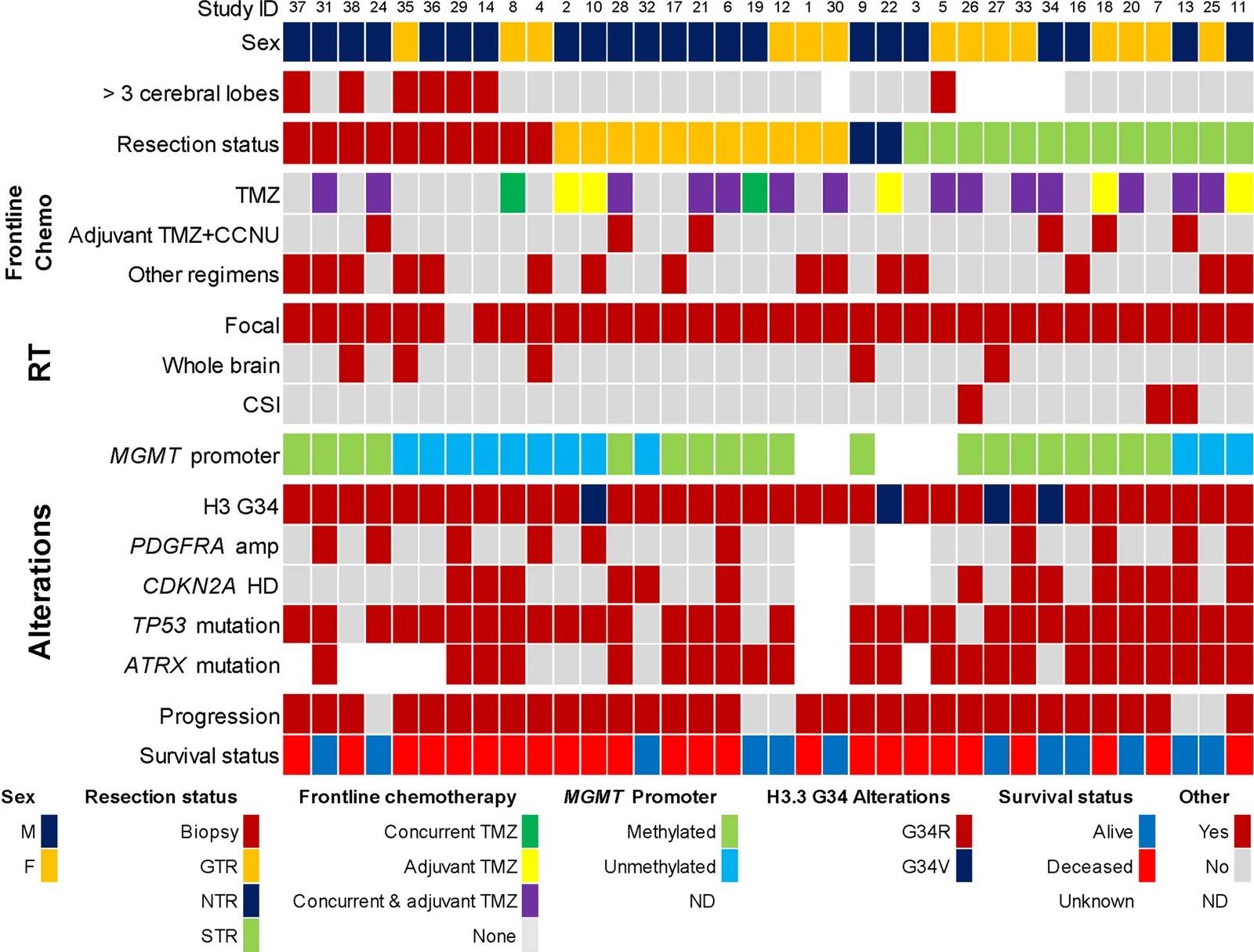
Clinical and molecular characteristics of the study cohort. *CSI* craniospinal irradiation, *GTR* gross total resection, *HD* homozygous deletion, *NTR* near-total resection, *ND* not determined, *RT* radiation therapy, *STR* subtotal resection, *TMZ* temozolomide

Our multi-institutional cohort consisted of 36 pediatric patients with H3 G34-mutant DHG. The demographic, clinical, and molecular characteristics of the study cohort are summarized in Fig. 1 and Supplementary Table 1. Supplementary Figure 1 shows the swimmer plot, which displays individual patients’ treatments, disease courses, and outcomes.

**Fig. 2:**
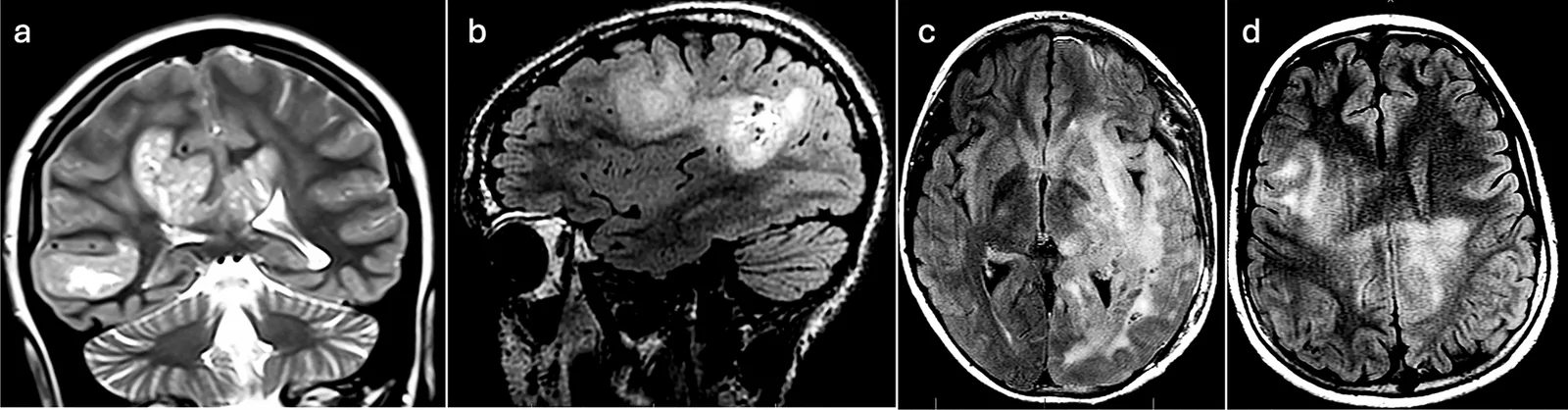
Representative images of four patients (**a**–**d**) with gliomatosis cerebri. **a** Coronal T2 image of patient #5 showed multilobar tumor involvement of the right temporal lobe, right occipital lobe, corpus callosum, and left cingulate. **b** Sagittal FLAIR image of patient #14 showed multilobar tumor involvement of the left frontal, parietal, and temporal lobes. **c** Transverse FLAIR image of patient #35 showed multilobar tumor involvement of the left basal ganglia, left thalamus, left side of the splenium, left frontal, left occipital, and left temporal lobes. **d** Transverse FLAIR image of patient #38 showed multilobar tumor involvement of the bilateral frontal, parietal lobes, corpus callosum, and the left splenium (not shown)

The median age was 14 years (range, 8–18 years). Twenty-two (61.1%) were male, and 14 (38.9%) were female. Tumor location was determined by central imaging review (*n* = 31) and from the clinic notes (*n* = 5). The maximum tumor dimensions ranged from 2.8 to 10.9 cm. Of the 31 patients with preoperative imaging available for review, imaging appearances were variable (Supplementary Fig. 2). Thirteen tumors (41.9%) involved more than one lobe at presentation, and seven tumors (22.6%) displayed a pattern of gliomatosis cerebri (Fig. 2).

*MGMT* promoter methylation was detected in 61.3% (19/31) of tumors using the MGMT-STP27 algorithm with available tissue for testing. *TP53* mutations are the most common concomitant molecular alterations, occurring in 88.2% (30/34) of tumors with available tissue for testing. Concomitant *ATRX* alterations occurred in 82.1% (23/28) of tumors with available tissue for testing. *CDKN2A* homozygous deletion was noted in 43.8% (14/32) of tumors. *PDGFRA* amplification was identified in 31.3% (10/32) of tumors, and mutations were identified in 3 tumors (total *n* = 13, 40.6%).

### Treatment and outcomes

Eleven patients (30.6%) had a GTR, two (5.6%) had an NTR, 13 (36.1%) had an STR, and 10 (27.8%) had a biopsy. Thirty five (97.2%) received upfront RT. One (2.8%) did not receive RT due to rapid disease progression. Of the patients who received upfront RT, 27 (77.1%) received only focal treatment, 3 (8.6%) received whole-brain RT (WBRT), and 3 (8.6%) received craniospinal irradiation (CSI). Two patients (5.7%) were noted to have symptomatic and radiographic progression during focal RT and were transitioned to WBRT.

Twenty-one (58.3%) patients received frontline TMZ, administered either concurrently with RT, as adjuvant therapy, or both. Of these 21 patients, six received adjuvant CCNU with TMZ.

**Fig. 3:**
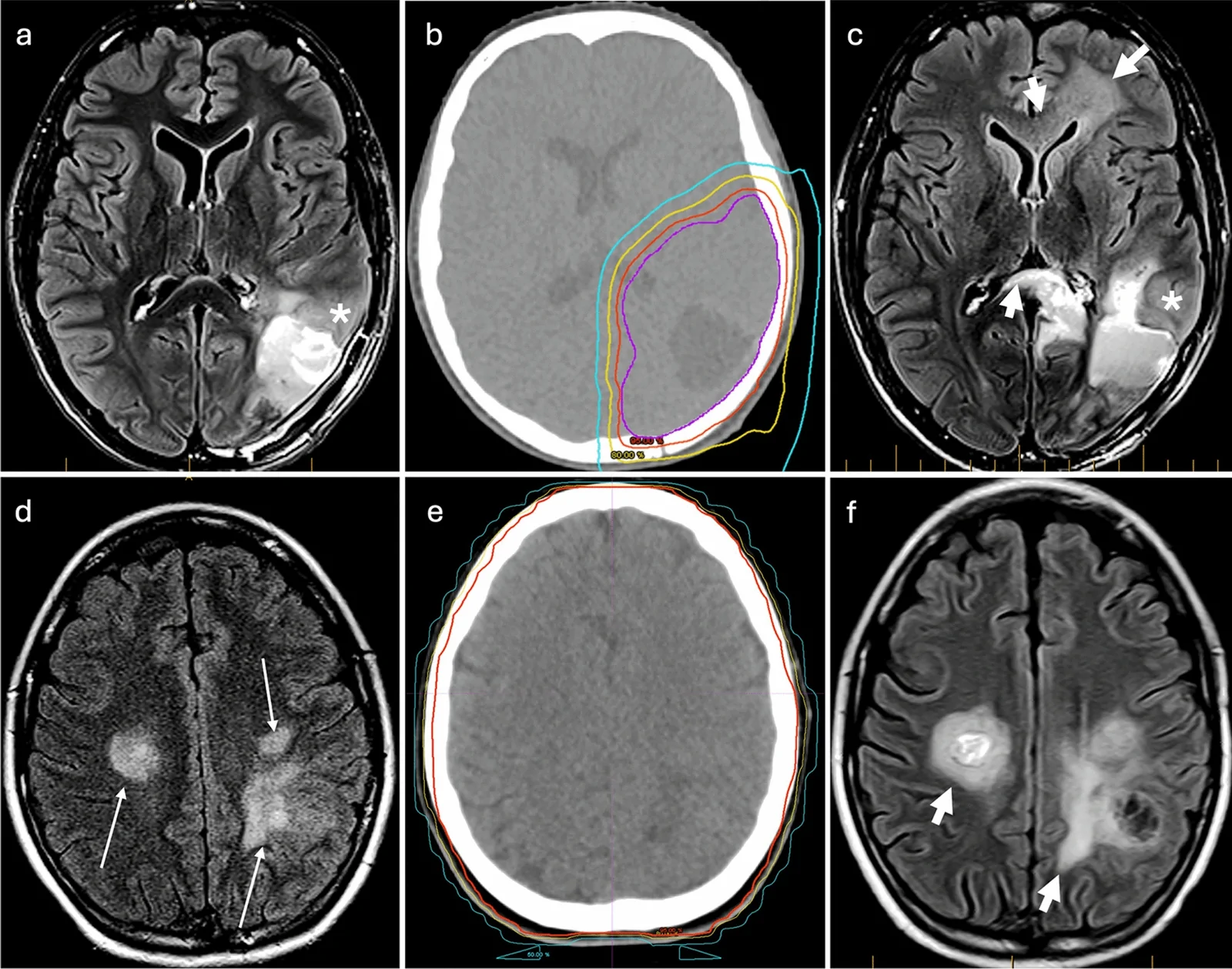
Patterns of recurrence in pediatric H3 G34-mutant DHG. (**a**–**c**, patient #11) Synchronous local and distal failure. **a** Axial T2-FLAIR image after partial resection demonstrates residual tumor anterior to the resection cavity in the right temporal lobe (asterisk). **b** The isodose lines of radiation planning are in the same plane as A. The patient received focal RT to a total dose of 59.4 Gy. The purple line shows the target volume; the red line shows the 95% dose line (56.4 Gy); the yellow line shows the 80% dose line (47.5 Gy); and the turquoise line shows the 50% dose line (29.7 Gy). The median splenium and genu of the corpus callosum and periventricular white matter around the left frontal horn are outside the 95% dose line. **c** Axial T2-FLAIR image on the same patient at recurrence at the same plane as in **a** and **b** demonstrates residual tumor anterior to the surgical resection cavity (*) and new ill-defined FLAIR abnormalities involving the splenium of the corpus callosum crossing the midline (thick arrow), within and adjacent to the 95% dose line, respectively, suggesting local failure. New ill-defined FLAIR abnormality in the genu of the corpus callosum and periventricular white matter around the left frontal horn (thick arrows), distal to the 95% dose line, suggesting distal failure. (**d**–**f**, patient #4) Local failure in a patient with gliomatosis cerebri developed after receiving 14.4 Gy focal radiation to the left parietal region. The patient was then transitioned to whole-brain radiation to 50.4 Gy (left parietal 64.8 Gy total). **d** Axial T2-FLAIR image demonstrates tumor in the left posterior frontal and parietal lobes as well as in the right posterior frontal lobe (thin arrows). **e** The isodose lines of radiation planning are in the same plane as **d**. The patient received whole-brain radiation to a total dose of 50.4 Gy. The red line shows the 95% dose line (47.9 Gy); the yellow line shows the 80% line (40.3 Gy); and the turquoise line shows the 50% line (25.2 Gy), all overlapping on the calvarium. The right frontal lobe received the same dose as the left frontal lobe. **f** Axial T2-FLAIR image of the same patient at the same plane as **d** and **e** at recurrence demonstrates further enlargement of the tumors in both the left posterior frontal and parietal lobes and the tumor within the right frontal lobe (thick arrows). As the patient received whole-brain radiation to a definitive dose, this progression was considered a local failure

The median follow-up was 2.1 years (range 0.4–5.1 years). Thirty-one (86.1%) had disease progression during the follow-up period, including one who progressed before receiving RT. Of the 21 patients who progressed following RT with evaluable radiographic progression and 3D radiation therapy dosimetry for central review, eight (38.1%) had an isolated local recurrence, four (19%) had distant recurrence only, and nine (42.9%) had combined local and distant recurrence (Fig. 3). Of those with local progression, 13/17 (76.5%) patients experienced a central local failure (i.e., 95% of the recurrent tumor volume was within the high-dose RT field). The cumulative RT dose ranged from 39 Gy (in 3 Gy fractions) to 64.8 Gy (in 1.8 Gy fractions); 7 (20%) patients received proton therapy.

**Fig. 4:**
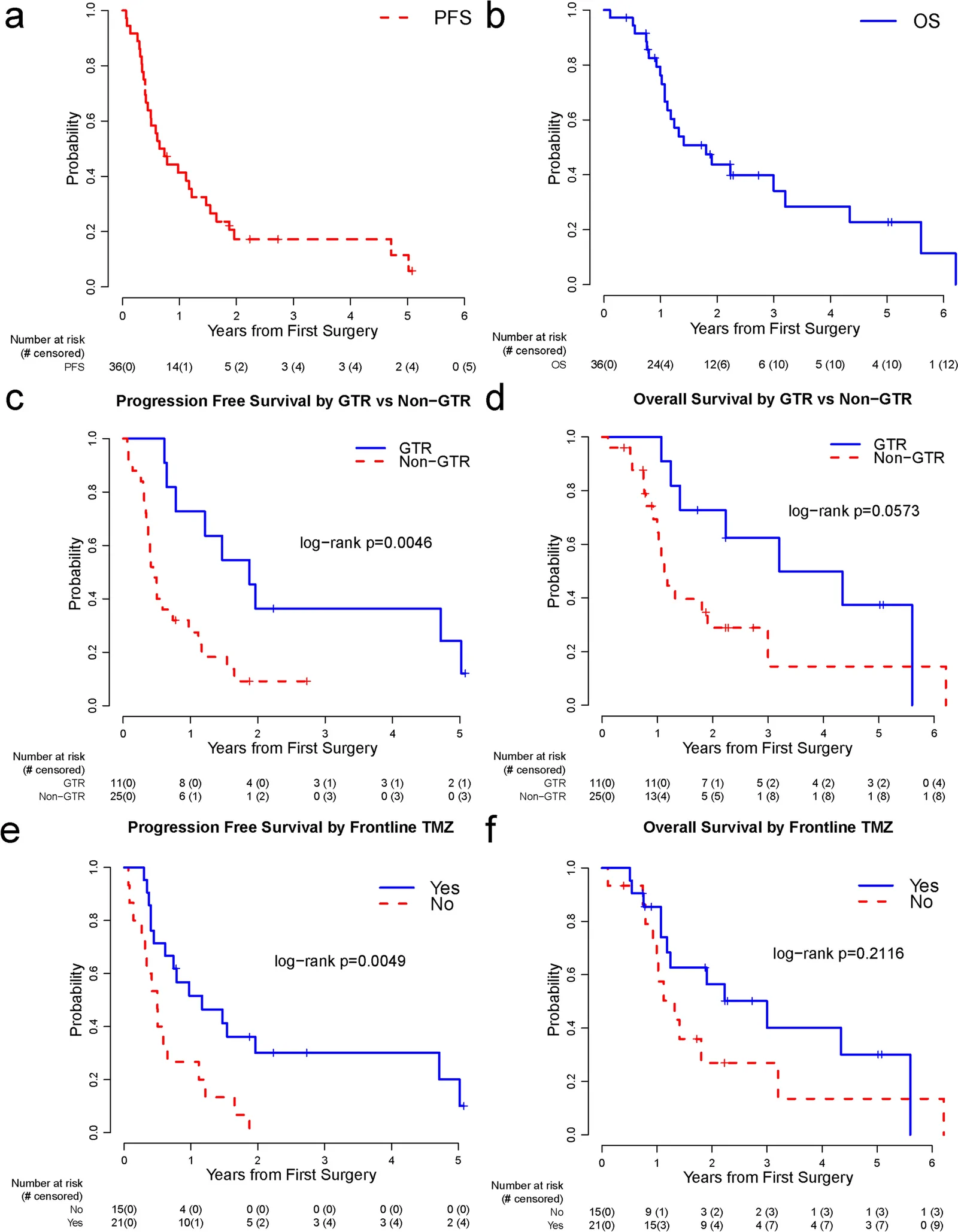
Kaplan–Meier survival analysis of clinical features in pediatric patients with H3 G34-mutant DHG. **a** PFS for all patients (*n* = 36), **b** OS for all patients (*n* = 36), **c** PFS by surgical resection status, **d** OS by surgical resection status, **e** PFS by frontline TMZ status, and **f** OS by frontline TMZ status. *PFS* progression-free survival, *OS* overall survival, *GTR* gross total resection, *TMZ* temozolomide

The PFS at 1, 2, and 5 years were 41.3% (95% CI 25.4–56.7%), 17.2% (95% CI 7.0–31.6%), and 11.5% (95% CI 2.9–27.1%), respectively. The median PFS was 0.7 years (95% CI 0.4–1.2 years) (Fig. 4a). The OS rates at 1, 2, and 5 years were 76.2% (95% CI 57.7–87.3%), 43.8% (95% CI 25.8–60.0%), and 22.7% (95% CI 8.3–41.6%), respectively. The median OS was 1.8 years (95% CI 1.1–3.2 years) (Fig. 4b). Patients who underwent GTR had significantly better PFS (*p* = 0.0046) (Fig. 4c), but the impact on OS was less pronounced compared to patients with non-GTR (Fig. 4d). Patients who received frontline TMZ had significantly better PFS (*p* = 0.0049) (Fig. 4e) but demonstrated less pronounced difference in OS compared to patients who did not receive frontline TMZ (Fig. 4f). Resection status and TMZ use were independent prognostic factors for PFS in our cohort, as determined by multivariate and Fisher’s exact tests (Supplementary Tables 2 and 3).

### Association of molecular markers with outcomes

**Fig. 5:**
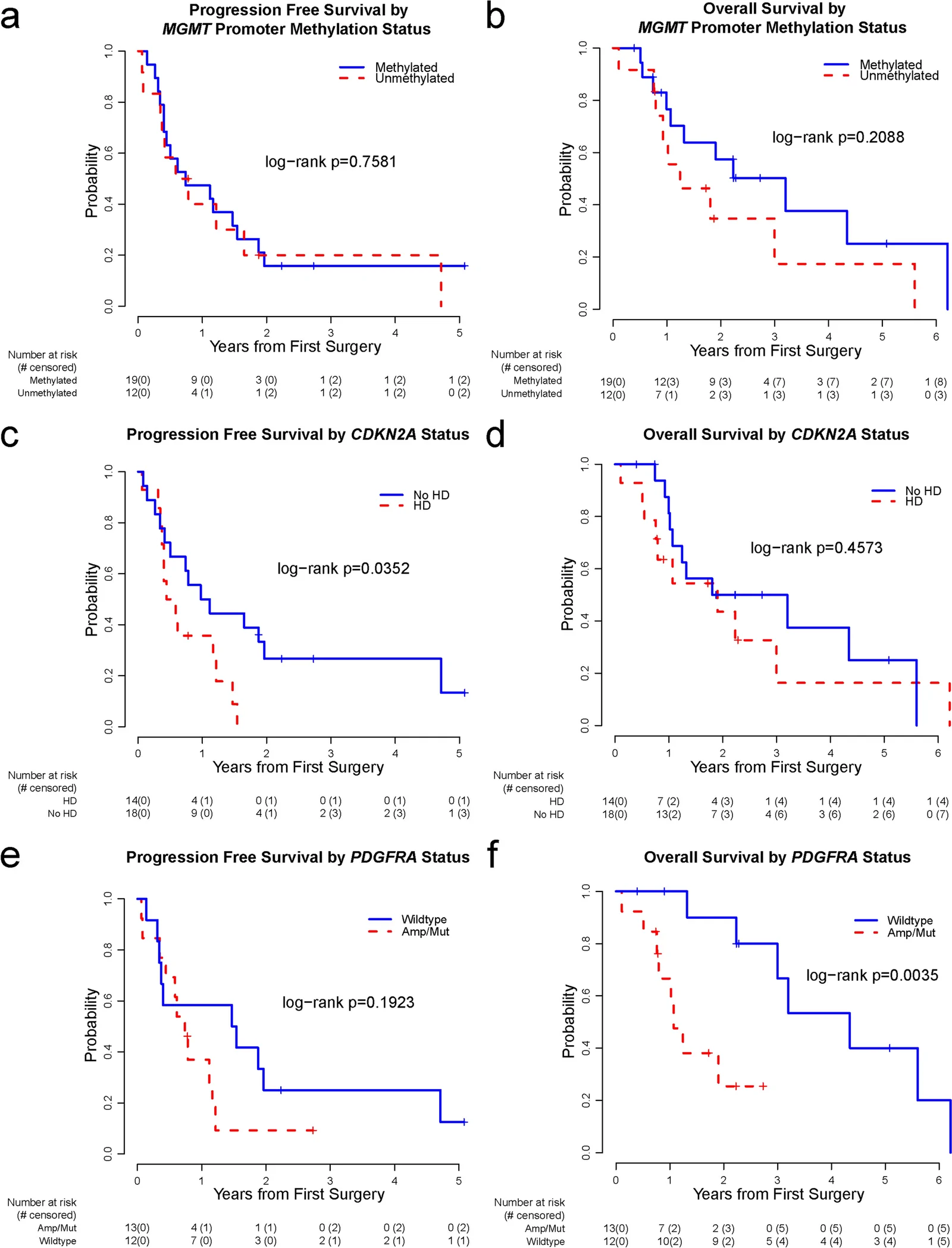
Kaplan–Meier survival analysis of molecular features in pediatric patients with H3 G34-mutant DHG. **a** PFS by *MGMT* promoter methylation status, **b** OS by *MGMT* promoter methylation status, **c** PFS by *CDKN2A* status, **d** OS by *CDKN2A* status, **e** PFS by *PDGFRA* status, and **f** OS by *PDGFRA* status. *PFS* progression-free survival, *OS* overall survival, *HD* homozygous deletion, *AMP* amplification

*MGMT* promoter methylation status, as determined by the MGMT-STP27 algorithm, was not associated with survival outcomes (Fig. 5a and b), and in patients receiving frontline TMZ, PFS and OS were not associated with *MGMT* promoter methylation status (*p* = 0.9073 for PFS and 0.9496 for OS). *CDKN2A* homozygous deletion was associated with significantly worse PFS (*p* = 0.0352) (Fig. 5c) but had no significant association with OS (Fig. 5d). *PDGFRA* amplification/mutation was not associated with worse PFS (Fig. 5e) but was associated with significantly worse OS (*p* = 0.0035) (Fig. 5f). There was no statistically significant association between *PDGFRA* amplification and GTR (OR 0.54, 95% CI 0.09–3.21; Fisher’s exact *p* = 0.68). *TP53* and *ATRX* mutation status, as well as the specific H3 G34 alteration (G34R vs. G34V), were not associated with patient outcomes.

**Fig. 6:**
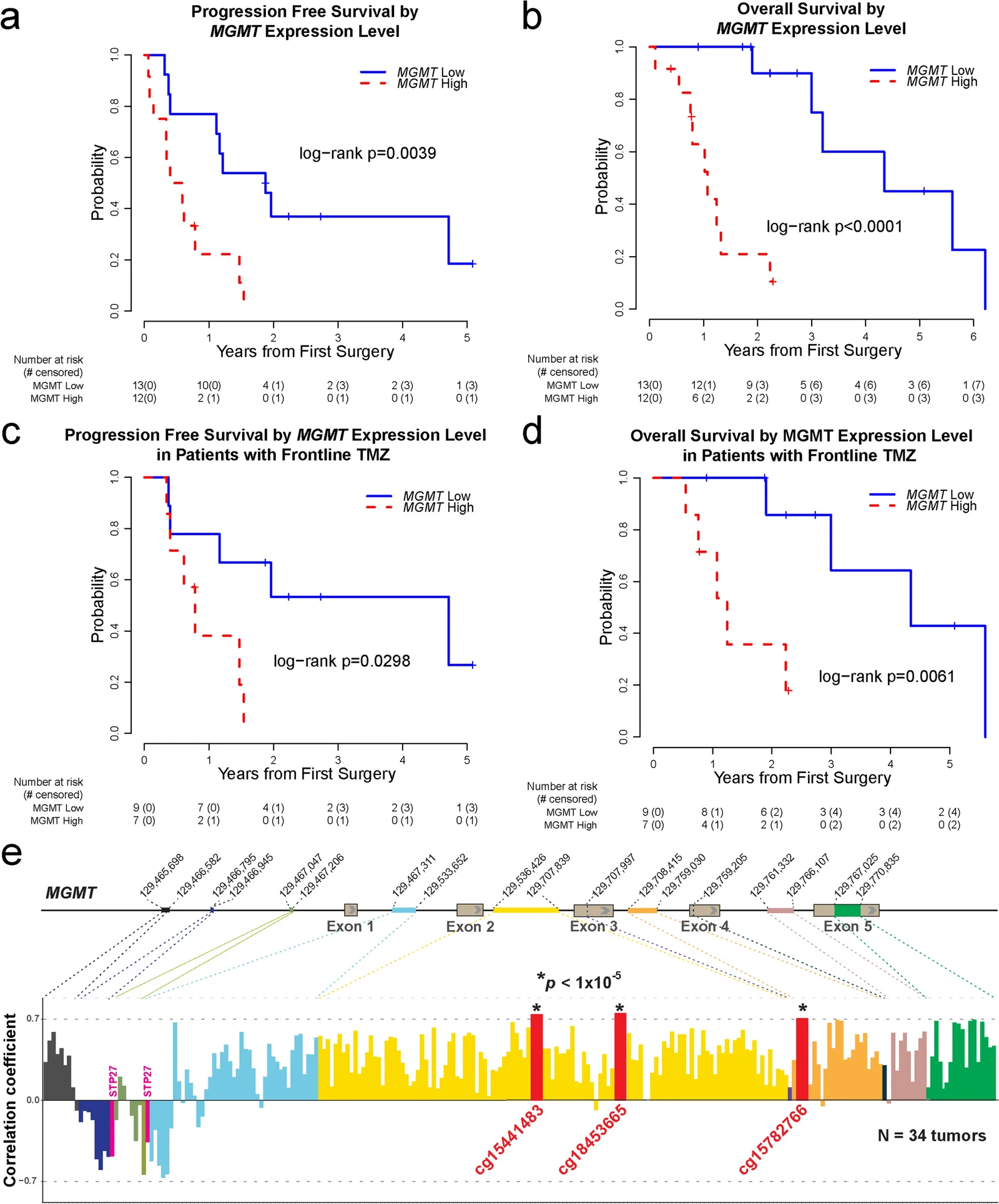
**a**–**d** Kaplan–Meier survival analysis of tumor *MGMT* mRNA expression levels in pediatric patients with H3 G34-mutant DHG. **a** PFS by *MGMT* expression levels, **b** OS by *MGMT* expression levels, **c** PFS by *MGMT* expression levels in patients with frontline TMZ, and **d** OS by *MGMT* expression levels in patients with frontline TMZ. **e** Correlation coefficients of specific CpG site at the *MGMT* locus with *MGMT* mRNA expression levels. *PFS* progression-free survival, *OS* overall survival, *GTR* gross total resection, *NTR* near-total resection, *STR* subtotal resection

We then sought to investigate the relationship between *MGMT* mRNA expression levels (Supplementary Table 4) and patient outcomes, as well as the association between tumor MGMT expression and outcomes in patients treated with TMZ. Low *MGMT* expression levels, with the median *MGMT* expression level as the cutoff, were associated with better PFS (*p* = 0.0039) (Fig. 6a) and OS (*p* < 0.0001) (Fig. 6b). Among patients who received TMZ, those with low *MGMT* expression levels in their tumors had improved PFS (*p* = 0.0298) (Fig. 6c) and OS (*p* = 0.0061) (Fig. 6d). There was no significant difference in survival outcomes between patients who did not receive TMZ and those who received TMZ and had high *MGMT* expression levels (Supplementary Fig. 3).

The discordant associations of *MGMT* promoter methylation status and *MGMT* mRNA expression levels with patient outcomes in the total and TMZ-treated cohorts prompted us to investigate other CpG sites at the *MGMT* locus that correlate with *MGMT* mRNA expression and, hence, potentially regulate *MGMT* gene expression. The DNA methylome and transcriptome data of 34 tumors were used for the analysis. Among the 293 probes on the Infinium EPIC v2.0 Array that cover the *MGMT* locus, 285 had sufficient quality for the correlation analysis (Fig. 6e). Three gene body/intronic CpG sites (cg15441483, cg18453665, and cg15782766) showed Spearman coefficients greater than 0.70 at statistically significant levels (*p* < 0.0001), indicating a strong positive correlation with *MGMT* mRNA expression. Probes cg12434587 and cg12981137 used by the MGMT-STP27 algorithm, on the other hand, had Spearman coefficients of − 0.48 and − 0.36, respectively. Analyses using the *β* values and *M *values for the methylation levels yielded similar results.

While the methylation levels (*β *values) of the three gene body/intronic CpG sites (cg15441483, cg18453665, and cg15782766) were not significantly associated with PFS (*p* = 0.0515, 0.0984, and 0.0694, respectively), the sum of the beta values was significantly associated with PFS (*p* = 0.0343). On the other hand, their individual and summed *β *values were significantly associated with OS (*p* = 0.0083, 0.0020, 0.0051, and 0.0013, respectively).

The presence of the I143V/K178R polymorphism in *MGMT* (in 5/22 tumors) and *MGMT* locus deletion (in 4/25 tumors) was not associated with patient outcomes.

### Multivariate analysis

To identify independent prognostic factors, we performed a multivariate Cox proportional hazards regression analysis including surgical resection status, TMZ use, gliomatosis cerebri growth pattern, *PDGFRA* amplification, and *CDKN2A* homozygous deletion status as the variables (Table 1).

**Table 1:** Multivariate Cox models with resection status, TMZ use, GC status, *PDGFRA* amplification, and *CDKN2A* homozygous deletion status as the variables

Survival outcome	Model variables	Hazard ratio	95% CI	Wald test *p* value
Progression-free survival	Resection (non-GTR vs. GTR)	6.38	1.16–34.98	0.0327
	TMZ (yes vs no)	0.12	0.02–0.68	0.0169
	GC (GC vs non-GC)	0.80	0.10–6.34	0.8320
	*PDGFRA* (no amplification vs amplification)	0.23	0.04–1.28	0.0934
	*CDKN2A* (wildtype vs homozygous deletion)	0.38	0.09–1.71	0.2084
Overall survival	Resection (non-GTR vs. GTR)	1.00	0.17–5.75	0.9999
	TMZ (yes vs no)	0.73	0.13–4.12	0.7260
	GC (GC vs non-GC)	228.4	5.92–8815.3	0.0036
	*PDGFRA* (no amplification vs Amplification)	0.02	0.002–0.28	0.0033
	*CDKN2A* (wildtype vs homozygous deletion)	1.73	0.33–9.04	0.5186

*GC* gliomatosis cerebri, *GTR* gross total resection, *TMZ* temozolomide

For PFS, surgical resection status and frontline TMZ use were again identified as independent prognostic factors. Non-GTR was significantly associated with an increased risk of progression (HR 6.38, 95% CI 1.16–34.98; *p* = 0.0327), while the use of frontline TMZ was associated with a reduced risk of progression (HR 0.12, 95% CI 0.02–0.68; *p* = 0.0169). The presence of gliomatosis cerebri (*p* = 0.8320), *PDGFRA* amplification (*p* = 0.0934), and *CDKN2A* homozygous deletion (*p* = 0.2084) were not significantly associated with PFS in the multivariate model. Consistent with these findings, univariate Kaplan–Meier analysis showed that patients with a gliomatosis cerebri pattern had a trend toward inferior PFS compared to those without, but this did not reach statistical significance (log-rank *p* = 0.0686) (Supplementary Fig. 4a).

For OS, the gliomatosis cerebri growth pattern and *PDGFRA* amplification status were identified as independent prognostic factors. The presence of a gliomatosis cerebri pattern was strongly associated with inferior OS (HR 228.4, 95% CI 5.92–8815.3; *p* = 0.0036), and the absence of *PDGFRA* amplification was significantly associated with improved OS (HR 0.02, 95% CI 0.002–0.28; *p* = 0.0033). Univariate analysis further corroborated the poor prognosis associated with a gliomatosis cerebri pattern, demonstrating significantly inferior OS for patients with gliomatosis cerebri (log-rank *p* < 0.0001) (Supplementary Fig. 4b). In contrast to the PFS results, neither surgical resection status (*p* = 0.9999) nor frontline TMZ use (*p* = 0.7260) was a statistically significant independent predictor of OS in the multivariate model.

## Discussion

H3 G34-mutant DHG is a devastating tumor type for which there is no curative therapy. Historically, the utilization of TMZ has been extrapolated from adult data and pediatric clinical trials that recruited patients with pHGG [10, 20, 39]. While TMZ is often incorporated into the treatment of these tumors in pediatrics, either as monotherapy or in combination with other agents, this practice remains inconsistent, and the standard of care remains surgery and RT [31]. Our study suggests that frontline TMZ confers PFS benefits to patients with H3 G34-mutant DHG, but it does not significantly impact OS. Multivariate analysis confirmed this observation, identifying frontline TMZ use as an independent prognostic factor significantly associated with improved PFS (HR 0.12, *p* = 0.0169). We could not draw conclusions about the utility of adding CCNU to this patient population, because only a limited number of patients in our cohort had received both CCNU and TMZ.

In adult HGG, *MGMT* promoter methylation testing is a well-established prognosticator, with a methylated *MGMT* promoter conferring a survival advantage and predicting benefit from TMZ [19]. In contrast, our analysis revealed no significant association between *MGMT* promoter methylation status and survival outcomes in pediatric patients with H3 G34-mutant DHG. Our study also showed no association between *MGMT* promoter methylation status and PFS or OS in patients receiving frontline TMZ, in distinction from adult data [19]. These findings do not align with the results of ACNS0126 [10] and some published experiences about H3 G34-mutant DHG [21, 23]. However, they are concordant with several pediatric studies, namely ACNS0423 [20], ACNS0822 [26], and the HERBY trial [28]. They also align with the H3 G34-mutant DHG meta-analysis results published by Vuong et al. [43].

To better understand the role of *MGMT* in pediatric H3 G34-mutant DHG, we evaluated *MGMT* mRNA expression in tumors. We found that low *MGMT* expression was associated with better PFS and OS and was significantly associated with improved outcomes in patients receiving frontline TMZ. This was an intriguing finding, given the lack of association between *MGMT* promoter methylation status and survival outcomes in the overall or TMZ-treated cohorts. We hypothesized that this discrepancy is related to a different mechanism of *MGMT* regulation in pediatric H3 G34-mutant DHG. Indeed, our analysis revealed that, rather than promoter methylation, gene body/intronic CpG methylation is significantly associated with *MGMT* mRNA expression levels in pediatric H3 G34-mutant DHG. Our work thus provides a mechanistic explanation for the lack of association between *MGMT* promoter methylation and outcomes. It also reveals the potential biological differences between adult tumors and pediatric tumors. To our knowledge, these findings have not been previously described in pediatrics and offer novel insights into this tumor type, with both prognostic and therapeutic implications. Our findings suggest that *MGMT* expression level may have prognostic value in pediatric H3 G34-mutant DHG. In addition, our findings do not support the use of *MGMT* promoter methylation status as a therapeutic guide in this patient population. A prospective study and a larger cohort are needed to further validate these findings.

In our cohort, we did not identify characteristic imaging features of H3 G34-mutant DHG, as appearances varied between patients. This finding aligns with the previous studies [22]. Our cohort demonstrated a high rate of multilobar involvement (41.9%) at presentation, with 22.6% exhibiting a gliomatosis cerebri growth pattern—features that are more frequently seen in adult populations [17]. The proportion of patients presenting with gliomatosis cerebri in our cohort exceeds the 8.2% incidence reported in adults with diffuse gliomas [37]. Multivariate analysis underscored the critical impact of this growth pattern. While GTR was an independent prognostic factor for PFS, gliomatosis cerebri emerged as a powerful independent predictor of inferior OS (HR 228.4, *p* = 0.0036). This divergence suggests that while maximal surgical resection significantly delays disease progression, the extensive, infiltrating nature of gliomatosis cerebri ultimately drives mortality. It is crucial to recognize that quality of life remains critical in this incurable tumor type. Thus, we advise careful surgical planning and making every effort to preserve eloquent brain areas and neurological function while seeking a maximal safe resection.

In our cohort, while most patients treated with RT received only focal radiation, a proportion received either WBRT (8.6%) or CSI (8.6%) due to extensive disease burden, and two patients required transition to WBRT after initiation of focal RT because of symptomatic and radiographic disease progression. Evaluation of disease progression patterns revealed that among patients with evaluable disease progression, more than 80% developed either a local-only recurrence or a combined local and distant recurrence. In contrast, fewer than 20% had an isolated distant recurrence. As most treatment failures occurred within the high-dose RT field, extended radiation fields are not justified.

In addition, we identified molecular prognostic markers in our cohort. In our multivariate model, *PDGFRA* amplification was identified as a significant independent prognostic factor for OS (*p* = 0.0033). While *CDKN2A* homozygous deletion was associated with inferior PFS in univariate analysis, it did not retain significance in the multivariate setting for either PFS or OS. This suggests that, among other clinical variables, *PDGFRA* status is a more robust driver of overall survival in this population. The prognostic implications of *PDGFRA* amplification are supported by prior literature [21, 42]. In contrast, the role of *CDKN2A* deletion remains more controversial, as Vuong et al. found no association between *CDKN2A* deletion and survival outcomes in their meta-analysis [43].

Limitations of this study include its retrospective design and modest sample size—constraints that reflect both the rarity of H3 G34-mutant DHG and our deliberate focus on pediatric patients. Variability in management across centers may further reduce power for subgroup analyses and temper generalizability beyond pediatric settings. Even so, the multi-institutional scope, centralized reviews, and integrated clinical–imaging–molecular evaluation strengthen confidence in the associations observed and yield practice-relevant insights. To address remaining uncertainties and advance care, harmonized international prospective studies and mechanistic investigations are needed to validate these findings, refine temozolomide-based strategies, and elucidate determinants of radio- and chemoresistance.

In the setting of childhood cancer, employing a global perspective is particularly important as more than 90% of children diagnosed with cancer reside in low- and middle-income countries (LMICs) [44]. For children and adolescents with central nervous system tumors in LMICs, access to quality multimodal care, including neurosurgical care, radiotherapy, and essential medications for cancer treatment, remains suboptimal [11, 30, 33, 35]. Based on our findings, we suggest that further consideration be given to including TMZ on the World Health Organization (WHO)’s list of essential anti-neoplastic medicines [49]. Ultimately, we believe that multi-institutional collaborations and prospective efforts are necessary to enhance our knowledge about this tumor type and improve outcomes in a patient population in dire need of hope.

## Conclusions

Based on our study results, we recommend assessing *MGMT* mRNA expression levels when feasible. We also support the use of TMZ as part of the frontline chemotherapy regimen for pediatric patients with H3 G34-mutant DHG, particularly when *MGMT* expression levels are low. We also recommend pursuing a GTR when reasonable and assessing *PDGFRA* status as important molecular prognostic markers for pediatric H3 G34-mutant DHG. Future prospective research should be used to validate the described mechanism of *MGMT* regulation and to explore novel therapeutic agents or combination therapies built upon a TMZ backbone to improve outcomes for this patient population. For instance, recent work by Liu et al. identified CDK4/6 as a critical vulnerability in H3 G34-mutant DHG [25]. This mechanistic insight has been translated into the clinic through the CONNECT TarGeT trial (NCT05843253), which includes a specific arm for H3 G34-mutant DHG combining the CDK4/6 inhibitor ribociclib with a backbone of radiotherapy and temozolomide. This trial design aligns with our finding that TMZ serves as an active and essential component of frontline therapy.

## Importance of the study

This multi-institutional study represents a comprehensive evaluation of pediatric H3 G34-mutant diffuse hemispheric glioma (DHG), integrating clinical, molecular, and imaging data from 36 patients. We identify key prognostic factors—the extent of resection, gliomatosis cerebri growth pattern, temozolomide (TMZ) use, and molecular alterations—that correlate with outcomes. We uncovered a novel mechanism of *MGMT* regulation, in which gene body methylation, rather than promoter methylation, correlates with *MGMT* expression, which is associated with outcomes. These findings challenge established paradigms and highlight the limitations of extrapolating data from adult gliomas to children. We describe a high frequency of gliomatosis cerebri patterns and central treatment failures, emphasizing the need for tailored surgical and radiation strategies. Our findings provide actionable recommendations for diagnostic workup and frontline therapy in pediatric H3 G34-mutant DHG and establish a foundation for future prospective studies.

## Supplementary Information

Below is the link to the electronic supplementary material.

Supplementary file1 (DOCX 18 KB)

Supplementary file2 (JPG 1155 KB)

Supplementary file3 (JPG 699 KB)

Supplementary file4 (JPG 388 KB)

Supplementary file5 (JPG 367 KB)

Supplementary file6 (DOCX 43 KB)

Supplementary file7 (DOCX 29 KB)

Supplementary file8 (DOCX 29 KB)

Supplementary file9 (DOCX 30 KB)

## Data Availability

The methylation and NGS data generated and analyzed by the study have been deposited in the St. Jude Cloud (https://stjude.cloud).
