# Supplementary material for: Pediatric H3 G34-mutant diffuse hemispheric glioma: clinical, imaging and molecular prognostic factors, MGMT expression, and temozolomide response
Source: Acta Neuropathol. 2026 Mar 2;151(1):22. doi: 10.1007/s00401-026-02992-w (PMC12953265; doi:10.1007/s00401-026-02992-w)
Supplement: Supplementary file 1 — Supplementary file1 (DOCX 18 KB) [file 401_2026_2992_MOESM1_ESM.docx]

**Supplementary Legends**

**Supplementary Figure 1** Swimmer plot depicting individual patients’ treatment, disease course, and outcome.

**Supplementary Figure 2** **Imaging features of H3 G34-mutant DHG** (a) Axial T2-weighted image demonstrates a round, well-defined, heterogeneously T2 hyper-intense mass in the right temporal lobe with minimal peritumoral edema. (b) Coronal T2-weighted sequence demonstrates a well-defined heterogeneously T2 hyper-intense mass centered in the cingulate gyri and corpus callosum, which also demonstrates no significant peritumoral edema. (c) Axial T2-weighted image demonstrates an ill-defined T2 hyperintense mass in the left posterior frontal and parietal lobe (arrow). (d) Axial T2-weighted image demonstrates an ill-defined thickening of the gyri in the right anterior temporal lobe (arrow). (e) Axial T2-weighted image demonstrated an ill-defined mass that involves the right frontal, temporal, and occipital lobes as well as the right thalamus with evidence of central heterogeneous T2 hypointensity due to intratumoral hemorrhage. (f) Axial T2-weighted image demonstrates an ill-defined mass involving the splenium of the corpus callosum and right posterior cingulate gyrus that also involves the right posterior frontal and occipital lobes. (g) Axial head CT demonstrates a focal intraparenchymal hemorrhage in the right frontal lobe with extra-axial extension. No underlying mass is evident. (h) Axial head CT demonstrates a focal intraparenchymal hemorrhage in the left temporal lobe. No underlying mass is evident.

**Supplementary Figure 3 Kaplan-Meier survival analysis** of (a) Progression-free survival, No frontline TMZ vs. Frontline TMZ with high *MGMT* expression levels, and (b) Overall survival, No frontline TMZ vs. Frontline TMZ with high *MGMT* expression levels.

**Supplementary Figure 4 Kaplan-Meier survival analysis** of (a) Progression-free survival, tumors presented as gliomatosis cerebri (GC) vs. not (non-GC), and (b) Overall survival, tumors presented as gliomatosis cerebri (GC) vs. not (non-GC).

**Supplementary Table 1 Study Cohort Information**

**Supplementary Table 2** **Multivariate Cox models with both resection status and TMZ use** Resection status and TMZ use were significantly associated with progression-free survival in a multivariate Cox model.

**Supplementary Table 3** Fisher’s exact test; resection status was not significantly associated with TMZ usage.

**Supplementary Table 4 *MGMT* mRNA levels**
