# Supplementary figures and images for: Pediatric H3 G34-mutant diffuse hemispheric glioma: clinical, imaging and molecular prognostic factors, MGMT expression, and temozolomide response
Source: Acta Neuropathol. 2026 Mar 2;151(1):22. doi: 10.1007/s00401-026-02992-w (PMC12953265; doi:10.1007/s00401-026-02992-w)

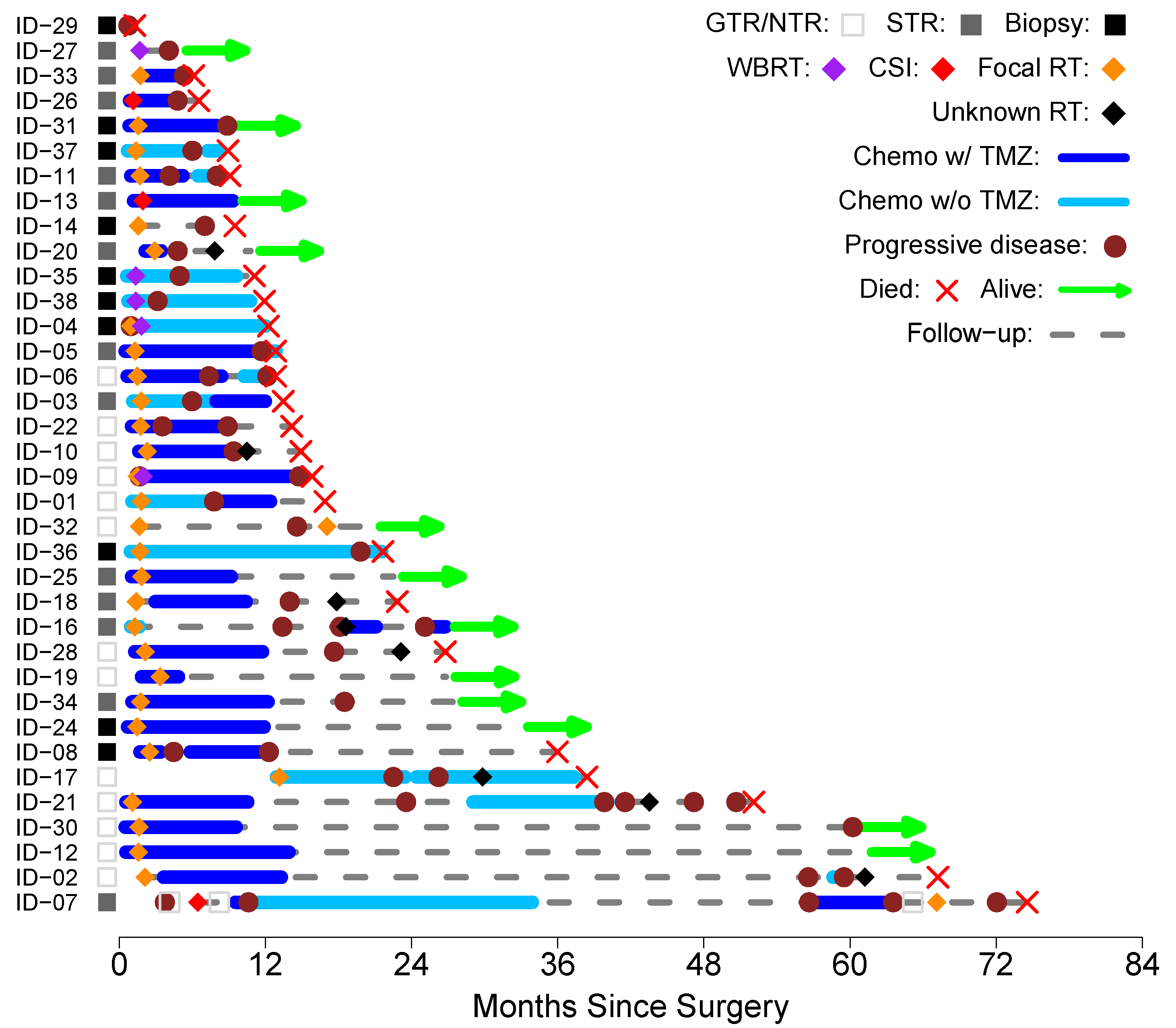

Supplement: Supplementary file 2 — Supplementary file2 (JPG 1155 KB) [file 401_2026_2992_MOESM2_ESM.jpg]

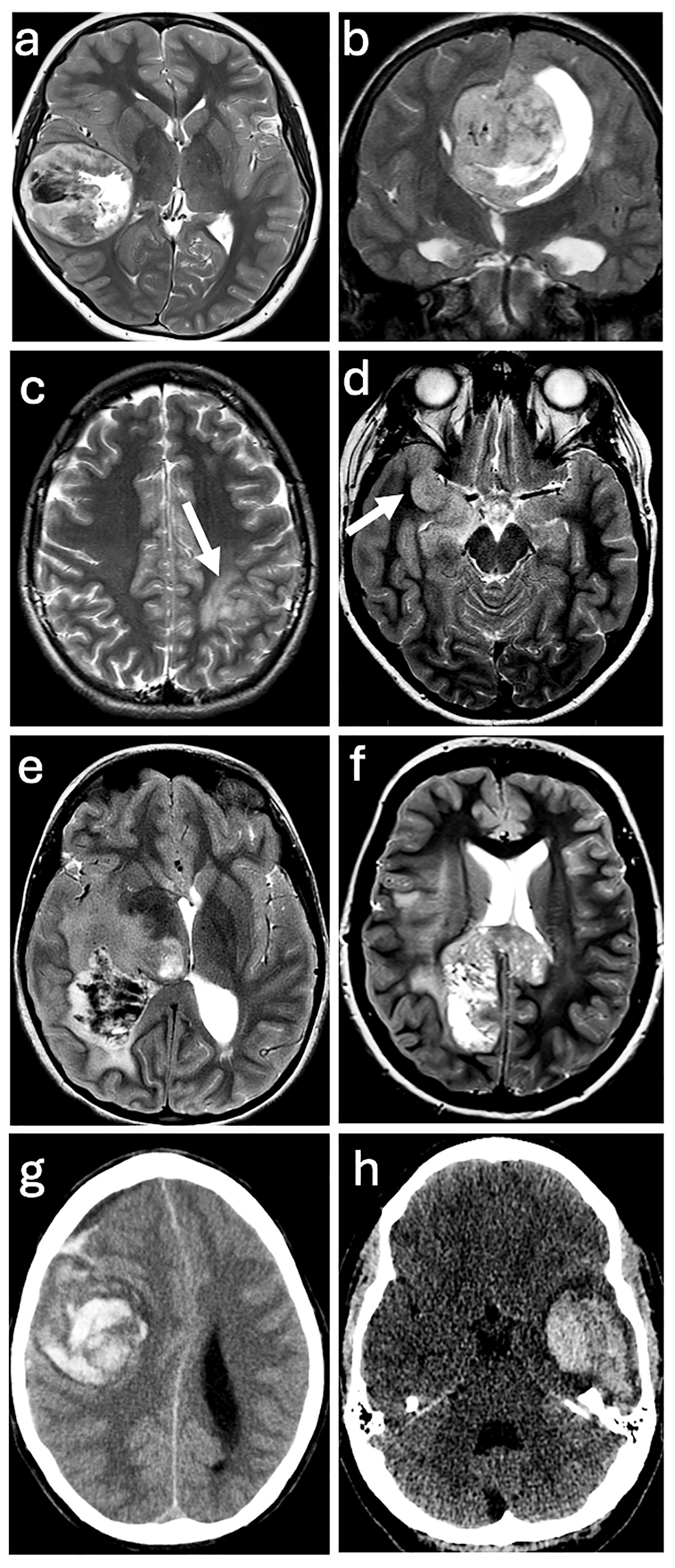

Supplement: Supplementary file 3 — Supplementary file3 (JPG 699 KB) [file 401_2026_2992_MOESM3_ESM.jpg]

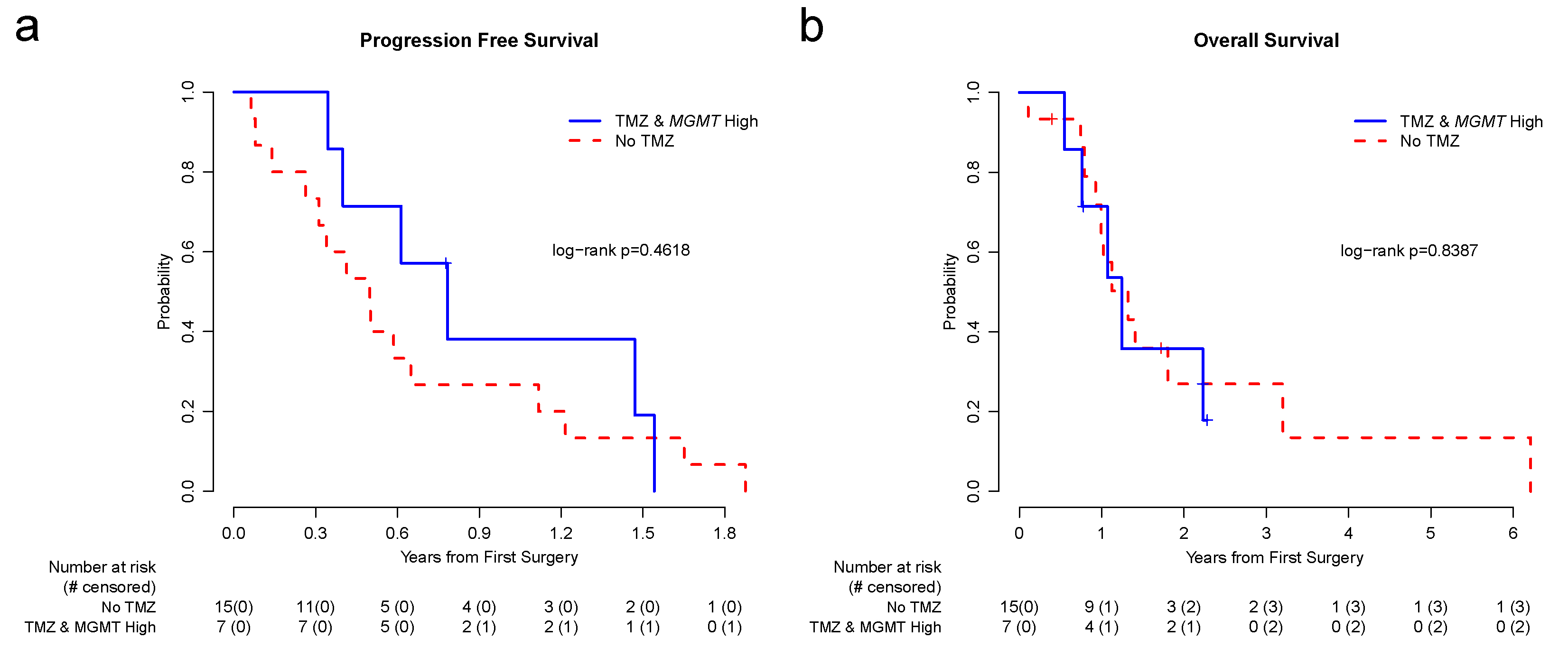

Supplement: Supplementary file 4 — Supplementary file4 (JPG 388 KB) [file 401_2026_2992_MOESM4_ESM.jpg]

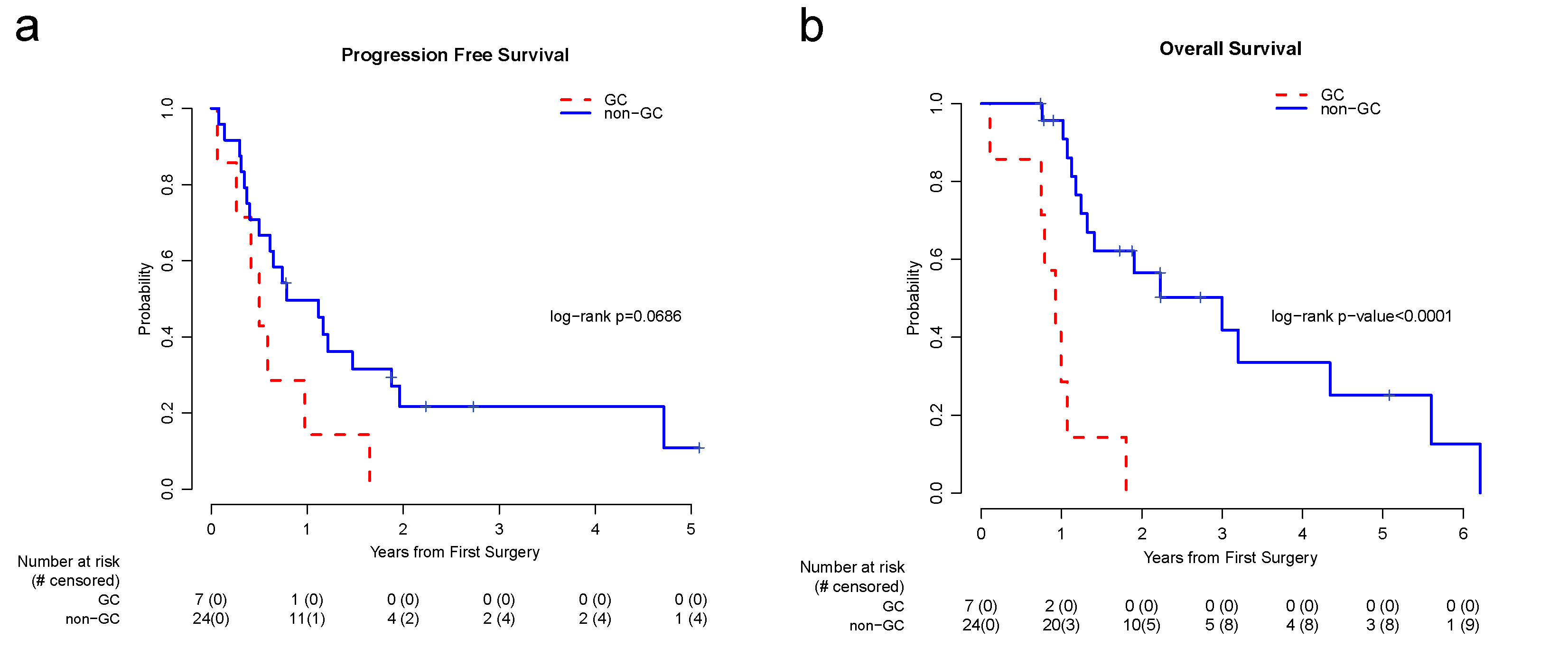

Supplement: Supplementary file 5 — Supplementary file5 (JPG 367 KB) [file 401_2026_2992_MOESM5_ESM.jpg]
