# Supplementary material for: Pediatric H3 G34-mutant diffuse hemispheric glioma: clinical, imaging and molecular prognostic factors, MGMT expression, and temozolomide response
Source: Acta Neuropathol. 2026 Mar 2;151(1):22. doi: 10.1007/s00401-026-02992-w (PMC12953265; doi:10.1007/s00401-026-02992-w)
Supplement: Supplementary file 6 — Supplementary file6 (DOCX 43 KB) [file 401_2026_2992_MOESM6_ESM.docx]

| **ID** | **Sex** | **GC^$^** | **Resection**  **Status** | **TMZ** | **CCNU+TMZ**  **Adjuvant** | **Other**  **regimens** | **RT** | ***MGMT***  **promoter** | **H3**  **G34** | **PDGFRA**  **amplification** | **CDKN2A**  **HD** | ***TP53***  **mutation** | ***ATRX***  **mutation** | **Progression** | **Survival**  **status** | **PFS**  **Censor*** | **PFS**  **(years)** | **OS**  **Censor*** | **OS**  **(years)** |
| --- | --- | --- | --- | --- | --- | --- | --- | --- | --- | --- | --- | --- | --- | --- | --- | --- | --- | --- | --- |
| 37 | M | Yes | Biopsy | None | No | Yes | Focal | Methylated | R | No | No | Yes | ND | Yes | Deceased | 1 | 0.5 | 1 | 0.74 |
| 31 | M | No | Biopsy | Concurrent & Adjuvant | No | Yes | Focal | Methylated | R | Yes | No | Yes | Yes | Yes | Alive | 1 | 0.74 | 0 | 0.74 |
| 38 | M | Yes | Biopsy | None | No | Yes | Focal, WBRT | Methylated | R | No | No | No | ND | Yes | Deceased | 1 | 0.26 | 1 | 0.99 |
| 24 | M | No | Biopsy | Concurrent & Adjuvant | Yes | No | Focal | Methylated | R | Yes | No | Yes | ND | No | Alive | 0 | 2.73 | 0 | 2.73 |
| 35 | F | Yes | Biopsy | None | No | Yes | Focal, WBRT | Unmethylated | R | No | No | Yes | ND | Yes | Deceased | 1 | 0.41 | 1 | 0.93 |
| 36 | M | Yes | Biopsy | None | No | Yes | Focal | Unmethylated | R | No | No | Yes | ND | Yes | Deceased | 1 | 1.65 | 1 | 1.8 |
| 29 | M | Yes | Biopsy | None | No | No | None | Unmethylated | R | Yes | Yes | Yes | Yes | Yes | Deceased | 1 | 0.06 | 1 | 0.11 |
| 14 | M | Yes | Biopsy | None | No | No | Focal | Unmethylated | R | No | Yes | Yes | Yes | Yes | Deceased | 1 | 0.59 | 1 | 0.79 |
| 8 | F | No | Biopsy | Concurrent | No | No | Focal | Unmethylated | R | No | Yes | Yes | Yes | Yes | Deceased | 1 | 0.37 | 1 | 3 |
| 4 | F | No | Biopsy | None | No | Yes | Focal, WBRT | Unmethylated | R | Yes | No | Yes | No | Yes | Deceased | 1 | 0.08 | 1 | 1.02 |
| 2 | M | No | GTR | Adjuvant | No | No | Focal | Unmethylated | R | No | No | Yes | No | Yes | Deceased | 1 | 4.71 | 1 | 5.6 |
| 10 | M | No | GTR | Adjuvant | No | Yes | Focal | Unmethylated | V | Yes | No | Yes | No | Yes | Deceased | 1 | 0.78 | 1 | 1.24 |
| 28 | M | No | GTR | Concurrent & Adjuvant | Yes | No | Focal | Methylated | R | No | Yes | Yes | Yes | Yes | Deceased | 1 | 1.47 | 1 | 2.23 |
| 32 | M | No | GTR | None | No | No | Focal | Unmethylated | R | No | Yes | No | No | Yes | Alive | 1 | 1.22 | 0 | 1.72 |
| 17 | M | No | GTR | None | No | Yes | Focal | Methylated | R | No | No | Yes | Yes | Yes | Deceased | 1 | 1.88 | 1 | 3.2 |
| 21 | M | No | GTR | Concurrent & Adjuvant | Yes | No | Focal | Methylated | R | No | No | Yes | Yes | Yes | Deceased | 1 | 1.96 | 1 | 4.34 |
| 6 | M | No | GTR | Concurrent & Adjuvant | No | No | Focal | Methylated | R | Yes | Yes | Yes | Yes | Yes | Deceased | 1 | 0.61 | 1 | 1.07 |
| 19 | M | No | GTR | Concurrent | No | No | Focal | Methylated | R | No | No | No | Yes | No | Alive | 0 | 2.23 | 0 | 2.23 |
| 12 | F | No | GTR | Concurrent & Adjuvant | No | No | Focal | Methylated | R | No | No | Yes | Yes | No | Alive | 0 | 5.08 | 0 | 5.08 |
| 1 | F | No | GTR | None | No | Yes | Focal | ND | R | ND | ND | ND | ND | Yes | Deceased | 1 | 0.65 | 1 | 1.41 |
| 30 | F | ND | GTR | Concurrent & Adjuvant | No | Yes | Focal | ND | R | ND | ND | ND | ND | Yes | Alive | 1 | 5.02 | 0 | 5.02 |
| 9 | M | No | NTR | None | No | No | Focal, WBRT | Methylated | R | No | No | Yes | Yes | Yes | Deceased | 1 | 0.14 | 1 | 1.32 |
| 22 | M | No | NTR | Adjuvant | No | Yes | Focal | ND | V | ND | ND | No | No | Yes | Deceased | 1 | 0.3 | 1 | 1.18 |
| 3 | M | No | STR | None | No | Yes | Focal | ND | R | ND | ND | Yes | ND | Yes | Deceased | 1 | 0.5 | 1 | 1.12 |
| 5 | F | Yes | STR | Concurrent & Adjuvant | No | No | Focal | ND | R | No | No | Yes | Yes | Yes | Deceased | 1 | 0.97 | 1 | 1.07 |
| 26 | F | ND | STR | Concurrent & Adjuvant | No | No | Focal, CSI | Methylated | R | No | Yes | No | Yes | Yes | Deceased | 1 | 0.4 | 1 | 0.54 |
| 27 | F | ND | STR | None | No | No | Focal, WBRT | Methylated | V | No | No | Yes | Yes | Yes | Alive | 1 | 0.34 | 0 | 0.39 |
| 33 | F | ND | STR | Concurrent & Adjuvant | No | No | Focal | Methylated | R | Yes | Yes | Yes | Yes | Yes | Deceased | 1 | 0.44 | 1 | 0.51 |
| 34 | M | ND | STR | Concurrent & Adjuvant | Yes | No | Focal | Methylated | V | No | Yes | Yes | No | Yes | Alive | 1 | 1.54 | 0 | 2.28 |
| 16 | M | No | STR | None | No | Yes | Focal | Methylated | R | No | No | Yes | Yes | Yes | Alive | 1 | 1.12 | 0 | 2.23 |
| 18 | F | No | STR | Adjuvant | Yes | No | Focal | Methylated | R | Yes | Yes | Yes | Yes | Yes | Deceased | 1 | 1.17 | 1 | 1.9 |
| 20 | F | No | STR | Concurrent & Adjuvant | No | No | Focal | Methylated | R | No | Yes | Yes | Yes | Yes | Alive | 1 | 0.4 | 0 | 0.9 |
| 7 | F | No | STR | None | No | No | Focal, CSI | Methylated | R | No | Yes | Yes | Yes | Yes | Deceased | 1 | 0.31 | 1 | 6.21 |
| 13 | M | No | STR | Concurrent & Adjuvant | Yes | No | Focal, CSI | Unmethylated | R | Yes | Yes | Yes | Yes | No | Alive | 0 | 0.78 | 0 | 0.78 |
| 25 | F | No | STR | Concurrent & Adjuvant | No | Yes | Focal | Unmethylated | R | No | No | Yes | Yes | No | Alive | 0 | 1.88 | 0 | 1.88 |
| 11 | M | No | STR | Adjuvant | No | Yes | Focal | Unmethylated | R | Yes | Yes | Yes | Yes | Yes | Deceased | 1 | 0.34 | 1 | 0.76 |

^$^>3 lobes; *0=censored, 1=event; CSI, craniospinal irradiation; GTR, gross total resection; HD: homozygous deletion; NTR, near-total resection; ND, not determined; RT, radiation therapy; STR, subtotal resection; TMZ, temozolomide
