# Supplementary material for: Pediatric H3 G34-mutant diffuse hemispheric glioma: clinical, imaging and molecular prognostic factors, MGMT expression, and temozolomide response
Source: Acta Neuropathol. 2026 Mar 2;151(1):22. doi: 10.1007/s00401-026-02992-w (PMC12953265; doi:10.1007/s00401-026-02992-w)
Supplement: Supplementary file 7 — Supplementary file7 (DOCX 29 KB) [file 401_2026_2992_MOESM7_ESM.docx]

| Survival Outcome | Model Variables | Hazard Ratio | 95% CI | Wald test p-value |
| --- | --- | --- | --- | --- |
| Progression-free survival | Resection (Non-GTR vs. GTR) | 3.17 | 1.30-7.71 | 0.0109 |
|  | Frontline TMZ (Yes vs No) | 0.38 | 0.18-0.81 | 0.0129 |
|  |  |  |  |  |
| Overall survival | Resection (Non-GTR vs. GTR) | 2.19 | 0.84-5.69 | 0.109 |
|  | Frontline TMZ (Yes vs No) | 0.73 | 0.31-1.72 | 0.469 |
