# Supplementary material for: Pediatric H3 G34-mutant diffuse hemispheric glioma: clinical, imaging and molecular prognostic factors, MGMT expression, and temozolomide response
Source: Acta Neuropathol. 2026 Mar 2;151(1):22. doi: 10.1007/s00401-026-02992-w (PMC12953265; doi:10.1007/s00401-026-02992-w)
Supplement: Supplementary file 8 — Supplementary file8 (DOCX 29 KB) [file 401_2026_2992_MOESM8_ESM.docx]

|  | **Received TMZ** | **No TMZ** | **Fisher’s Exact P-value** |
| --- | --- | --- | --- |
| **Resection status** |  |  |  |
| GTR | 8 | 3 | 0.2951 |
| Non-GTR | 13 | 12 |  |
