# Supplementary material for: Pediatric H3 G34-mutant diffuse hemispheric glioma: clinical, imaging and molecular prognostic factors, MGMT expression, and temozolomide response
Source: Acta Neuropathol. 2026 Mar 2;151(1):22. doi: 10.1007/s00401-026-02992-w (PMC12953265; doi:10.1007/s00401-026-02992-w)
Supplement: Supplementary file 9 — Supplementary file9 (DOCX 30 KB) [file 401_2026_2992_MOESM9_ESM.docx]

| **Study ID** | **MGMT Expression (log2)** |
| --- | --- |
| 2 | 0.8194 |
| 4 | 7.2806 |
| 6 | 9.3195 |
| 7 | 1.3245 |
| 8 | 1.5533 |
| 9 | 2.1416 |
| 10 | 8.8047 |
| 11 | 5.2283 |
| 12 | 1.0519 |
| 13 | 19.2531 |
| 14 | 3.9269 |
| 15 | 7.2896 |
| 16 | 0.4248 |
| 17 | 1.0053 |
| 18 | 1.0619 |
| 19 | 0.3024 |
| 20 | 0.6585 |
| 21 | 1.6070 |
| 23 | 1.2296 |
| 24 | 0.2517 |
| 25 | 0.4965 |
| 26 | 5.9790 |
| 27 | 6.3852 |
| 28 | 1.8143 |
| 29 | 25.9401 |
| 32 | 1.6765 |
| 34 | 4.6101 |
